# Over-Expression of a Cytochrome P450 Is Associated with Resistance to Pyriproxyfen in the Greenhouse Whitefly *Trialeurodes vaporariorum*

**DOI:** 10.1371/journal.pone.0031077

**Published:** 2012-02-08

**Authors:** Nikos Karatolos, Martin S. Williamson, Ian Denholm, Kevin Gorman, Richard H. ffrench-Constant, Chris Bass

**Affiliations:** 1 Rothamsted Research, Harpenden, Hertfordshire, United Kingdom; 2 Biosciences, University of Exeter, Penryn, United Kingdom; Ghent University, Belgium

## Abstract

**Background:**

The juvenile hormone mimic, pyriproxyfen is a suppressor of insect embryogenesis and development, and is effective at controlling pests such as the greenhouse whitefly *Trialeurodes vaporariorum* (Westwood) which are resistant to other chemical classes of insecticides. Although there are reports of insects evolving resistance to pyriproxyfen, the underlying resistance mechanism(s) are poorly understood.

**Results:**

Bioassays against eggs of a German (TV8) population of *T. vaporariorum* revealed a moderate level (21-fold) of resistance to pyriproxyfen. This is the first time that pyriproxyfen resistance has been confirmed in this species. Sequential selection of TV8 rapidly generated a strain (TV8pyrsel) displaying a much higher resistance ratio (>4000-fold). The enzyme inhibitor piperonyl butoxide (PBO) suppressed this increased resistance, indicating that it was primarily mediated via metabolic detoxification. Microarray analysis identified a number of significantly over-expressed genes in TV8pyrsel as candidates for a role in resistance including cytochrome-P450 dependent monooxygenases (P450s). Quantitative PCR highlighted a single P450 gene (*CYP4G61*) that was highly over-expressed (81.7-fold) in TV8pyrsel.

**Conclusion:**

Over-expression of a single cytochrome P450 gene (*CYP4G61*) has emerged as a strong candidate for causing the enhanced resistance phenotype. Further work is needed to confirm the role of the encoded P450 enzyme CYP4G61 in detoxifying pyriproxyfen.

## Introduction

Insecticide resistance in crop pests usually arises via one of two types of mechanisms: either reduced binding of the insecticide to its target through mutation of the target site (e.g. acetylcholinesterase for organophosphates/carbamates, the voltage-gated sodium channel for pyrethroids and the nicotinic acetylcholine receptor for neonicotinoid insecticides) [1], or increased detoxification or sequestration of insecticides [1], [2] by enzymes such as carboxylesterases (CEs) [3], glutathione-S-transferases (GSTs) [4] and cytochrome P450-dependent monooxygenases [5].

Pyriproxyfen (2-[1-methyl-2-(4-phenoxyphenoxy)-ethoxy] pyridine) is a juvenile hormone analogue (JHA) effective against some arthropod pests including the greenhouse whitefly *Trialeurodes vaporariorum* Westwood and the sweet potato whitefly, *Bemisia tabaci* Gennadius (Hemiptera: Aleyrodidae). Pyriproxyfen is a potent suppressor of embryogenesis and later development that competes for juvenile hormone receptor binding sites and disrupts the transition from one developmental stage to another [6]–[8]. The mode of action of pyriproxyfen is not fully understood due to the lack of a known signalling pathway and/or a receptor molecule. However, the *Methoprene-tolerant* gene (*Met*) (also known as *Resistance to juvenile hormone*) has been proposed as a possible candidate for the juvenile hormone (JH) receptor as it has been shown to confer resistance to toxic doses of JH when mutated [9], [10].

Resistance to pyriproxyfen was first documented in *B. tabaci* from Israel in 1998 [11], [12] and early studies suggested that P450s were not involved in the catabolism of pyriproxyfen [13]. However, more recent biochemical work on laboratory selected strains from Arizona indicated that P450s and GSTs were involved in pyriproxyfen detoxification [14]. To date, resistance to this compound has not been described in *T. vaporariorum*, an important virus vector and pest of protected vegetable and ornamental crops in temperate regions of the world [15], [16] that has developed resistance to numerous other chemical classes including pyrethroids and neonicotinoids [17]–[20].

The aim of the present study was to investigate potential mechanisms of pyriproxyfen resistance in a laboratory selected strain of *T. vaporariorum* exhibiting over 4000-fold resistance to pyriproxyfen. 454-based pyrosequencing has recently been used to provide a substantial expressed sequence tag (EST) data-set containing over 50,000 sequence contigs for *T. vaporariorum* [21]. We have used this as a reference transcriptome for cDNA microarray design and then to identify candidate genes that are associated with the resistance phenotype.

## Results and Discussion

### Bioassays

**Figure 1:**
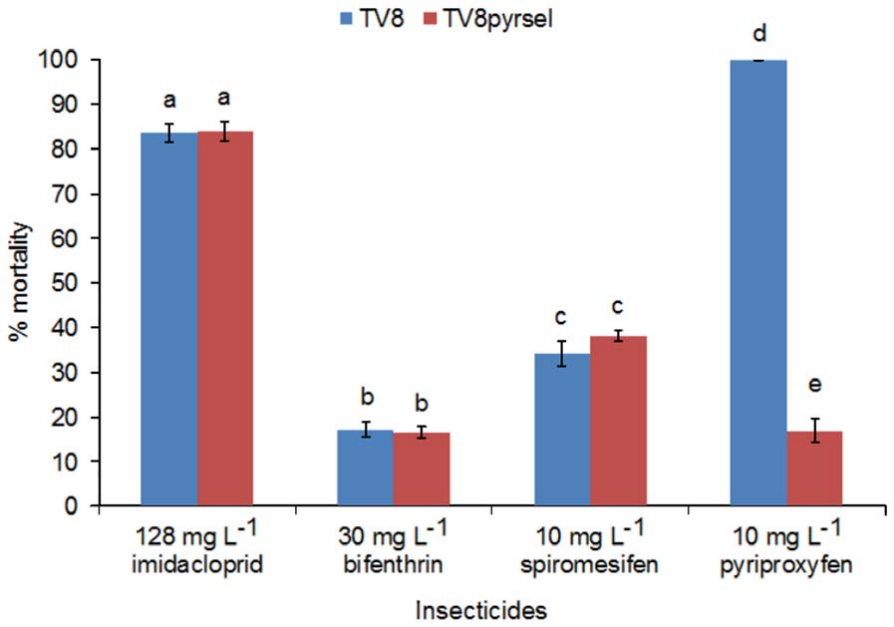
Percentage mortality of diagnostic doses of imidacloprid (neonicotinoid), bifenthrin (pyrethroid), spiromesifen (tetronic acid derivative) and pyriproxyfen in the selected strain TV8pyrsel and the unselected parental strain TV8. Different letters indicate significant differences between strains based on a two-sample unpaired t-test.

The UK strain TV3 and the German strain TV8 showed 5- and 21-fold resistance to pyriproxyfen, respectively (Table 1). Selection of TV8 with pyriproxyfen increased resistance to 4,574-fold compared to TV1 and 223-fold compared to the unselected TV8. These findings provide the first confirmation of pyriproxyfen resistance in *T. vaporariorum*. The response of TV8pyrsel after only three generations of selection demonstrated a very potent resistance to this insecticide. Interestingly, pyriproxyfen is not registered for use in Germany. The moderate resistance found in TV8 could be due to either cross-resistance between pyriproxyfen and a different class of insecticides or transfer of whitefly infested plant materials from regions were pyriproxyfen is used for whitefly control. However, selection of TV8 with pyriproxyfen did not result in enhanced resistance to other compounds belonging to major insecticides classes used for whitefly control, such as neonicotinoids, tetronic acid derivatives and pyrethroids (Figure 1). This leads to the conclusion that the resistance identified in this strain is due to European or global plant trade.

**Table 1:** Responses of *Trialeurodes vaporariorum* eggs to pyriproxyfen and synergism effect of pyriproxyfen after pre-treatment with PBO to the susceptible TV1 and the pyriproxyfen selected strain TV8pyrsel.

Insecticide	Strain	LC_50_ (mg L^−1^) (95% CL)*	Slope	RF
pyriproxyfen	TV1	0.014 (0.012–0.016) a	2.05 (±0.11)	1
pyriproxyfen+PBO	TV1	0.010 (0.009–0.011) a	2.15 (±0.10)	1
pyriproxyfen	TV3	0.05 (0.04–0.06) b	1.21 (±0.06)	5
pyriproxyfen	TV8	0.29 (0.26–0.31) c	1.89 (±0.08)	21
pyriproxyfen	TV8PyrSel	63.9 (58.3–70.2) d	2.96 (±0.13)	4574
pyriproxyfen+PBO	TV8PyrSel	0.22 (0.18–0.27) c	1.94 (±0.11)	21

Resistance factors (RFs) relative to TV1 are given for all the field strains.

*Different letters indicate significant difference between strains, based on overlapping 95% CL of LC_50_ values.

Pre-treatment of TV8pyrsel with the enzyme inhibitor piperonyl butoxide (PBO) reduced resistance to the level found in the pre-selected strain (TV8). There was no equivalent synergism of pyriproxyfen by PBO in the susceptible strain TV1 (Table 1). This result provided strong evidence that pyriproxyfen resistance in TV8pyrsel is primarily due to enhanced detoxification by either cytochrome P450 monooxygenases or CEs (both enzyme families are inhibited by PBO). This hypothesis was investigated further using microarrays and quantitative PCR.

### Microarray and quantitative real-time PCR analyses

Microarray analysis identified 3,474 probes (5.5% of the probes which corresponded to 3,227 unique contigs) as significantly differentially transcribed between the pyriproxyfen selected strain TV8pyrsel and the susceptible standard TV1 (Figure S1). These genes along with Log2, calculated fold-change values and closest BLAST hits are listed in Table S1. 1,865 probes (1,032 corresponding to genes with unknown function) had elevated expression in TV8pyrsel and 1,609 (1,105 of unknown function) were down-regulated relative to TV1. Of the 833 over-expressed probes with a known function, 25 were identified as potential candidates for causing insecticide resistance (Table 2). These included probes corresponding to genes encoding cytochrome P450s (19), CEs (3), GSTs (3), enzymes that have been implicated in insecticide resistance in many arthropod species [3]–[5].

**Table 2:** Selected metabolic genes identified by microarray as differentially transcribed between the pyriproxyfen resistant *Trialeurodes vaporariorum* strain TV8pyrsel and the susceptible TV1.

Contig number	Family/gene name^1^	Probe Name	Fold change	Log2	Hit accession
8639	P450 *CYP6DT4*	CUST_4671_PI425265390	7.53	2.91	ACT68012
31414	P450 *CYP6DS1*	CUST_4667_PI425265390	6.58	2.72	ACT68012
5194	P450 *CYP6CM4*	CUST_4669_PI425265390	6.40	2.68	ACD84797
4648	P450 *CYP4G61*	CUST_1785_PI425308678	6.00	2.59	XP_001944205
21292	P450 *CYP4G61*	CUST_7415_PI425265390	5.59	2.48	XP_001944205
13018	P450 *CYP18A1*	CUST_4064_PI425308678	5.36	2.42	XP_002427451
13018	P450 *CYP18A1*	CUST_6265_PI425265390	5.29	2.40	XP_002427451
41451	P450 *CYP6DT6*	CUST_50026_PI425265390	5.16	2.37	ACT68012
41451	P450 *CYP6DT6*	CUST_3539_PI425308678	5.04	2.33	ACT68012
16136	P450 *CYP314A1*	CUST_5821_PI425308678	5.05	2.34	XP_001948607
16136	P450 *CYP314A1*	CUST_10486_PI425265390	4.98	2.32	XP_001948607
5866	P450 *CYP4G60*	CUST_7412_PI425265390	4.25	2.09	XP_001944205
9000	P450 *CYP6DT7*	CUST_49998_PI425265390	3.91	1.97	CAZ65617
45973	P450 *CYP6DT5*	CUST_4692_PI425265390	3.17	1.67	ACT68012
50476	P450 *CYP6DP2*	CUST_50025_PI425265390	2.53	1.34	CAH65682
41100	P450 *CYP6DP2*	CUST_4690_PI425265390	2.48	1.31	CAH65682
50476	P450 *CYP6DP2*	CUST_3538_PI425308678	2.44	1.29	CAH65682
4672	P450 *CYP353C1*	CUST_2054_PI425308678	2.11	1.08	EFA01331
4672	P450 *CYP353C1*	CUST_49588_PI425265390	2.08	1.06	EFA01331
5401	CE clade A	CUST_51868_PI425265390	3.26	1.71	ABV45410
11569	CE clade A	CUST_15720_PI425265390	2.98	1.58	XP_001663733
4777	CE clade A	CUST_51886_PI425265390	2.53	1.34	XP_392698
11236	microsomal gst	CUST_54255_PI425265390	4.05	2.02	XP_002428068
263	microsomal gst	CUST_54254_PI425265390	2.02	1.01	XP_002428068
7168	delta gst	CUST_13266_PI425265390	2.49	1.32	EFA01955
17998	CE clade A	CUST_52068_PI425265390	−2.79	−1.48	EFA06762
42539	P450 *CYP306A1*	CUST_7409_PI425265390	−2.74	−1.46	XP_001600763

The family/gene names of known genes, as well as accession of the top blast hits are given [21].

1 Cytochrome P450 names were given by Dr. David Nelson [39].

Twelve gene sequences (Table 2) encoding cytochrome P450s (19 probes) were elevated in TV8pyrsel (2.08–7.53 fold). In six cases duplicate probes (generated either for the same contig or for allelic variant of the same contig) corresponding to the same P450 gene (*CYP4G61* of the CYP4 family, *CYP6DT6* and *CYP6DP2* of the CYP3, *CYP18A1* of the CYP2, and the mitochondrial P450s *CYP314A1* and *CYP353C1*) were over-expressed in TV8pyrsel (2.08–6 fold). In other insect pests, members of the CYP2, CYP3 and CYP4 microsomal P450 families are most commonly implicated in the metabolism of synthetic insecticides [5]. Microsomal P450s have been implicated in pyriproxyfen resistance in the house fly, *Musca domestica* L. (Diptera: Muscidae) [22], [23], the yellow fever mosquito, *Aedes aegypti* L. (Diptera: Culicidae) [24] and the whitefly, *B. tabaci* [14]. In the house fly, P450s were shown to metabolise pyriproxyfen into two major metabolites; 4′-OH-pyr and 5″-OH-pyr [22]. In the TV8pyrsel strain of *T. vaporariorum*, a total of seven genes belonging to the CYP3 family, two to the CYP4 family, and one to the CYP2 family were over-expressed. In *B. tabaci*, it was shown that pyriproxyfen treatment in a resistant strain induces expression of the cytochrome P450 *CYP9F2* gene [25]. However, no close ortholog of this gene was over-expressed in *T. vaporariorum*.

Three sequences encoding CEs (contigs 5401, 11569 and 4777), all of them belonging to clade A [21] were identified as being over-expressed in the resistant strain (Table 2). The level of expression of these sequences was moderate (2.53–3.26-fold) and the proteins these genes encode are therefore unlikely to be playing a significant role in resistance to pyriproxyfen. Three sequences encoding GSTs (contigs 7168, 11236 and 263) were elevated in the resistant strain (Table 2). Of these contig 7168 belongs to the delta class, members of which have been shown to be associated with insecticide resistance. However, this sequence was elevated by only 2.49-fold in the resistant strain.

Of the 504 probes with a known function that were down-regulated in TV8pyrsel, only two detoxification genes were identified (Table S1; Table 2). These included one contig (42539) encoding a cytochrome P450 (*CYP306A1*) with a negative fold change of −2.74 (0.36-fold) and a single sequence encoding a CE (belonging to clade A) with a fold change of −2.79 (0.36-fold). These genes were selected to validate the microarray data by quantitative PCR.

Real-time quantitative PCR was used to validate the microarray results by examining the expression profile of nine selected P450 genes (8 over-expressed and 1 down-regulated in the resistant strain), one CE that was found to be down-regulated in TV8pyrsel and finally two housekeeping genes (*EF1a* and *para*). For each housekeeping gene, data were normalised using the other as a reference. In most cases, the over- or under-transcription of the genes was confirmed (Table 3), although expression ratios obtained from RT-PCR were frequently different from those generated by microarray. Discrepancies in the data obtained from microarray experiments using the Agilent array platform and real-time quantitative RT-PCR have been described previously [26]–[28] and our results again highlight the importance of RT-PCR validation of array results.

**Table 3:** Fold change in expression of selected metabolic enzymes (P450s and a carboxylesterase (CE)), *EF1a*, and *para* in the pyriproxyfen resistant *Trialeurodes vaporariorum* strain TV8pyrsel (compared to the standard susceptible strain TV1) determined by quantitative PCR and microarray technology.

Gene name	contig number	Fold change - microarray	Fold change compared to TV1 - q pcr (95% CL)	
			TV8	TV8pyrsel
*CYP6DT4*	8639	7.53	1.19 (0.96–1.42)	1.20 (1.07–1.33)
*CYP6DS1*	31414	6.58	1.45 (1.43–1.46)	1.29 (0.98–1.59)
*CYP6CM4*	5194	6.40	0.99 (0.97–1.02)	1.15 (0.97–1.33)
*CYP4G61*	21292, 4648	5.59–6.00	1.42 (0.69–2.15)	81.7 (81.6–81.9)
*CYP4G60*	5866	4.25	1.24 (0.91–1.57)	1.14 (0.98–1.30)
*CYP18A1*	13018	5.29–5.36	1.09 (0.90–1.29)	2.54 (2.29–2.78)
*CYP6DT6*	41451	5.04–5.16	1.13 (0.98–1.28)	1.43 (1.32–1.55)
*CYP6DT7*	9000	3.91	1.59 (0.86–2.32)	1.12 (1.09–1.14)
*CYP6DT5*	45973	3.17	1.54 (1.13–1.95)	1.15 (0.93–1.37)
CE Tv17998	17998	0.36	0.51 (0.33–0.69)	0.75 (0.68–0.81)
*CYP306A1*	42539	0.36	0.31 (0.12–0.51)	0.44 (0.13–0.74)
*EF1a* 1	1983	0.93–1.17	0.81 (0.53–1.08)	0.94 (0.32–1.56)
*para* 1	21272, 22691, 37637	1.04–1.58	1.24 (0.98–1.50)	1.06 (0.44–1.68)

1 For each housekeeping gene, data were normalised using the other one as a reference.

Of the candidate genes encoding detoxification enzymes examined by RT-PCR only a single P450 gene (*CYP4G61*) was found to be highly over-expressed in TV8pyrsel displaying an 81.7-fold increase in transcription. The significantly lower expression level obtained from the microarray data for this gene may be partially explained by the well-known underestimation of expression ratios by microarrays compared with RT-PCR [29]. The expression of the *CYP4G61* gene in the original unselected field strain TV8 was also examined by RT-PCR (Table 3). The expression ratio of this gene in this strain compared to TV1 was 1.41-fold (0.66–2.16) indicating that the enhanced expression of this gene in the highly resistant strain TV8pyrsel is a result of sequential selection with pyriproxyfen. q-PCR using a second primer pair (cyp4g61-f', cyp4g61-r') confirmed these findings with the expression ratio of the unselected TV8 being 1.52-fold (1.39–1.65) and that of TV8pyrsel being 94.1 (93.8–94.4).

Members of the CYP4G cytochrome P450 subfamily have been shown to be involved in insecticide detoxification in other insect species. Examples are *CYP4G8* and *CYP4G19* which are involved in pyrethroid detoxification in the cotton bollworm, *Helicoverpa armigera* Hübner (Lepidoptera: Noctuidae) [30] and the German cockroach, *Blattella germanica* Linnaeus (Blattodea: Blattellidae) [31] respectively. Two other genes of this family are known to be induced after treatments with insecticides; *CYP4G36*, induced by imidacloprid in *A. aegypti* [32] and *CYP4G2*, induced by permethrin in *M. domestica* [33]. The *CYP4G61* in *T. vaporariorum* shares 66% amino acid identity with *CYP4G8* (AAD33077), 60% with *CYP4G36* (EAT44585), 56% with *CYP4G19* (AAO20251), 48% with *CYP4G2* (ABV48808).

### *CYP4G61* copy number

It has been recently shown that the enhanced transcription of a cytochrome P450 gene (*CYP6CY3*) in a resistant clone of peach potato aphid, *Myzus persicae* Sulzer (Hemiptera: Aphididae) is due to structural amplification of the gene [28]. Quantitative PCR was used to determine *CYP4G61* gene copy number using genomic DNA from individual adult male whiteflies (haploids) as template. Data were normalised using two genes; *para* (present in a single copy in insects as revealed by several genome sequencing projects [34]) and *EF1a* (present in two copies in Hymenoptera and Diptera, but in a single copy in most other insect species [35]). The mean cycle threshold values of three biological replicates in quantitative PCR of the *CYP4G61*, *EF1a* and *para* genes were essentially the same in all strains (for TV1 CTs of 27.5, 27.6 and 27.7 respectively, for TV8 27.3, 27.5 and 27.7, and for TV8pyrsel 27.1, 27.5 and 27.5) indicating that haploid *T. vaporariorum* males carry a single copy of the *CYP4G61* gene. Neither the field strain TV8 or the pyriproxyfen selected TV8pyrsel showed any significant fold increase compared to TV1. TV8 showed a fold change of 1.08 (0.56–1.60) and 1.14 (0.86–1.42) compared to the TV1 using *EF1a* or *para* to normalise respectively. Similarly, TV8pyrsel showed a fold increase of 1.25 (0.35–2.14) and 1.19 (0.67–1.71) compared to the TV1. These results indicate that the increased expression of the *CYP4G61* gene likely arises through mutation of *cis*-acting promoter sequences and/or *trans*-acting regulatory loci [36] rather than gene amplification.

### *CYP4G61* cDNA characterization

Two allelic variant contig sequences representing the *CYP4G61* gene were identified in the InsectaCentral database (http://insectacentral.org) and manually curated [21]. These were contig 21292 (partial sequence, assembled by 28 454-reads) and contig4648 (full length sequence, assembled by 106 454-reads). These contigs were assembled from reads from two cDNA libraries, one for the susceptible strain TV1 and the other for a neonicotinoid resistant strain. These libraries were tagged prior to sequencing using molecular markers [21]. An initial analysis of these assemblies (after reassembling them from the related ESTs) revealed the presence of 10 silent single nucleotide polymorphisms (SNPs) at nucleotide positions 126, 435, 774, 867, 966, 1146, 1329, 1356, 1620 and 1653 (Figure S2). There were only two substitutions, which cause an amino acid change; one was a G/C at amino acid position 282 that causes an amino acid substitution of a glycine to an alanine (G/A) and an A/T at position 395 that causes an amino acid substitution of a serine to cysteine (S/C) (Figure S2). The complete mRNA includes a 5′ UTR of 164 bp and a 3′ UTR of 270 bp.

**Figure 2:**
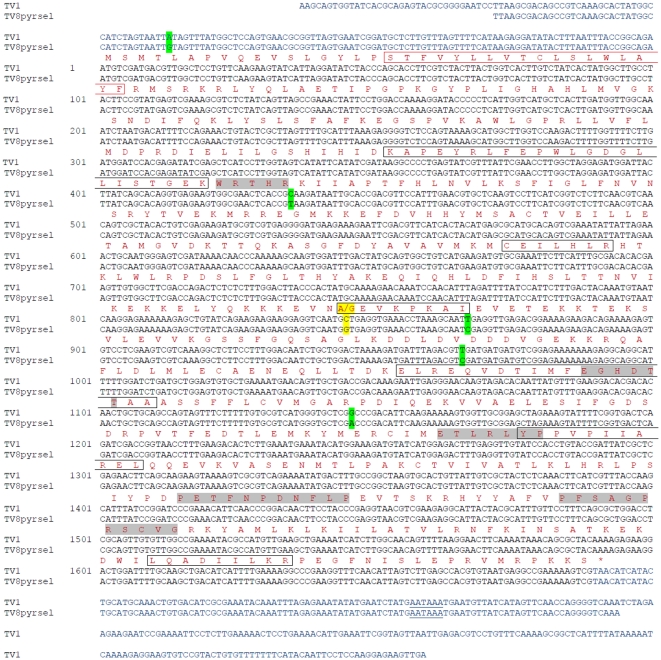
Complete cDNA sequence of the *CYP4G61* gene of *Trialeurodes vaporariorum* for the strains TV1 and TV8pyrsel. Conserved domains common to cytochrome P450s are highlighted in grey. These are the helix C motif, the helix I motif, the helix K motif, the PERF motif and the heme-binding “signature” motif. 5′ and 3′ UTR sequences are representing with light blue text. The polyadenylation signal, AATAAA is underlined. Sites of SNPs are colour marked: green for silent or UTR SNPs and yellow for amino-acid substitutions. Marked in boxes are the N-terminal transmembrane anchor (red-line box) and the SRS 1–6 (black-line box).

The cDNA contains a 1689 bp open reading frame (Figure 2) encoding 563 amino acid residues, with a calculated molecular mass of 64,449 kDa and a predicted isoelectric point of 8.65. The encoded protein contains conserved domains common in cytochrome P450s, such as the helix C motif (WxxxR; position 141), helix I (oxygen binding) motif ([A/G]Gx[E/D]T[T/S]; position 363), the helix K motif (ExxRxxP; position 421), the PERF motif (PxxFxP[E/D]RF; position 472) and the heme-binding “signature” motif (PFxxGxxxCxG; position 495). The polyadenylation signal AATAAA is located 73 nucleotides downstream of the 3′ end coding region.

Variations in the coding sequence of *CYP4G61* in two strains of *T. vaporariorum* (TV1 and TV8pyrsel; Figure S3) were investigated by either an analysis of the reassembled sequence for the strain TV1 (for which there was excellent sequence coverage) or by direct nucleotide sequencing for TV8pyrsel. In TV1, nine polymorphic sites were identified (nucleotide positions 126, 435, 774, 845, 867, 966, 1146, 1356, 1620), but only one change (C/G) at amino acid position 282 results in an amino acid substitution of an alanine to a glycine (A/G) (Figure 2). Only one polymorphism (C/T) for this strain appeared to occur in a conserved protein motif (helix C), but this was a synonymous substitution. For TV8pyrsel the coding sequence was much more conserved than TV1, probably as a result of selection, and only two silent polymorphisms in nucleotide positions 126 and 774 were identified. After comparing the consensus cDNA sequence of TV1 and TV8pyrsel, four synonymous SNPs at positions 145 (CGC/CGT), 289 (ATT/ATC), 322 (GTT/GTC), and 382 (CGG/CGA) and one non-synonymous SNP at position 282 (GCT/GGT) conferring an alanine to glycine (A/G) substitution were observed. Interestingly, the latter appeared to be at the beginning of the SRS 3. Finally, a single SNP (A/G) was found in the 5′UTR of the *CYP4G61* (Figure 2).

### Conclusions

Based on the results of this study, *CYP4G61* emerges as the strongest candidate for further investigation into its role in conferring potent resistance to pyriproxyfen in *T. vaporariorum*. In particular, functional characterisation of this P450 to confirm its ability to detoxify pyriproxyfen is now required.

## Materials and Methods

### Insect strains

Three strains of *T. vaporariorum* including an insecticide susceptible reference strain (TV1) were used in this study (Table 4). All were reared at Rothamsted Research without exposure to insecticides on French bean plants, *Phaseolus vulgaris* L., cv. “Canadian Wonder” (Fabaceae), under a 16 h photoperiod at 24°C.

**Table 4:** *Trialeurodes vaporariorum* strains, origins, year of collection and host.

Strain	Country of origin	Year of collection	Original host
TV1	UK	1971	French bean
TV3	UK	2008	Ornamentals
TV8	Germany	2008	Ornamentals

### Insecticides and bioassays

Pyriproxyfen 0.5 G was obtained as a commercial formulation (Sumilarv®; Sumitomo Chemical Corporation) and diluted to the required concentrations in distilled water containing 0.1 g L^−1^ of the non-ionic wetter Agral® (Syngenta). Technical piperonyl butoxide (PBO) (PCP ‘Ultra’) was provided by Dr. Graham Moores (Rothamsted Research).

The responses of strains TV1 (susceptible standard strain), TV3 and TV8 to pyriproxyfen were determined using a leaf-dip bioassay method modified to measure egg-hatch suppression. Leaves on intact bean plants were cut into rectangles of approximately 40 mm×50 mm. These plants were placed in cages with at least 200 adult whiteflies for 24 h to obtain a synchronised cohort of eggs, after which the adults were removed. Egg infested leaves were dipped for 15 seconds in the required concentration of insecticide or into 0.1 g L^−1^ Agral® as a control. Treated plants were maintained at 24°C and mortality was scored after 11 days by counting un-hatched eggs and live nymphs. Concentration-mortality relationships were fitted by probit analysis, using the software GenStat 12^th^ edition (VSN International Ltd, Hertfordshire, UK). Resistance factors were calculated by dividing LC_50_ values for field strains by that for the susceptible standard (TV1). Lack of overlap of 95% confidence limits on fitted LC_50_ values denoted significant differences in response. For the synergism bioassays, whitefly eggs were initially dipped into a 0.1% PBO solution in acetone followed 5 h later by insecticide as described above.

Insects of TV8 were selected for resistance by treating eggs for three successive generations with 3 mg L^−1^, 5 mg L^−1^, and 10 mg L^−1^ pyriproxyfen, respectively, to generate a selected strain denoted TV8PyrSel. In order to investigate for patterns of cross-resistance, the pyriproxyfen selected and the unselected parental strain were tested with diagnostic doses of the neonicotinoid imidacloprid 200 g L^−1^ SL (Confidor®; Bayer CropScience), the pyrethroid bifenthrin 100 g L^−1^ EC (Gyro®, CERTIS) and the tetronic acid derivative spiromesifen 240 g L^−1^ SC (Oberon®; Bayer CropScience).

### Microarray design

A SurePrint G3 (8×60 k) expression array was designed using Agilent's eArray platform. The base composition and the best probe methodologies were selected to design sense orientation 60-mer probes with a 3′ bias. The recently published *T. vaporariorum* EST assembly (54,751 contigs) [21] was used as the reference transcriptome. 60-mer probes were designed for all 54,751 assembled contigs, including contigs encoding detoxification enzymes (P450s, GSTs and CEs). Additional probe groups for 15 plant genes of *Phaseolus vulgaris* Linnaeus (Fabales: Fabaceae) for negative controls and a default set of Agilent controls were also included. The array was filled to capacity using alternate 60-mer probes for a selection of the *T. vaporariorum* contigs that returned a blast result in the nr database [21]. The final slide layout consists of 8 arrays of 62,976 elements. This array design can be made available (and ordered) by third parties on request through a shared work space set up on eArray. Table S2 provides information about probes and corresponding contigs, as well as a description of the top BLAST hit in the NCBI nr database for each contig (note that only descriptions of contigs with a BLAST result are shown in this file).

This microarray was used to compare gene expression in the highly selected pyriproxyfen resistance strain TV8PyrSel with the susceptible standard strain TV1. Total RNA was extracted from four pools of approximately 500 whitefly eggs, using the Isolate RNA Mini Kit (Bioline) according to the manufacturer's protocol. 830 ng of each total RNA was used to generate labelled cRNA, which was hybridized to arrays and these were washed and scanned as described in Agilent's Quick Amp Labelling Protocol (Version 6.5). The microarray experiment consisted of four biological replicates and for each of these, two hybridisations were done in which the Cy3 and Cy5 labels were swapped between samples for a total of eight hybridisations between resistant and susceptible strains.

Microarrays were scanned with an Agilent G2505C US10020348 scanner, and fluorescent intensities of individual spots were obtained using the Agilent Feature Extraction software with default Agilent parameters. Data normalization, filtering, dye flipping and statistical analysis were performed using the GeneSpring GX suit. For statistical analysis, a t-test against zero using the Benjamini-Hochberg false discovery rate (FDR) method for multiple testing corrections was used to detect significantly differentially expressed genes. Genes meeting a p value cut-off of 0.01 and showing a transcription ratio >2 fold in either direction were considered to be differentially transcribed between the two strains. All microarray data were MIAME compliant and they were submitted to the Gene Expression Omnibus (GEO) database with accession number GSE31316.

### Quantitative RT–PCR

Quantitative RT-PCR was used to validate microarray data by examining the expression profile of 14 genes (primarily ones encoding P450s) chosen on the basis of their likelihood as candidates for causing resistance. Primers were designed to amplify a fragment of 90–150 bp in size and are listed in Table S3. Total RNA was prepared as described before and four micrograms was used for cDNA synthesis using Superscript III and random hexamers (Invitrogen) according to the manufacturer's instructions. PCR reactions (20 µl) contained 4 µl of cDNA (10 ng), 10 µl of SensiMix SYBR Kit (Bioline), and 0.25 mM of each primer. Samples were run on a Rotor-Gene 6000 (Corbett Research) using the temperature cycling conditions of: 10 minutes at 95°C followed by 40 cycles of 95°C for 15 s, 57°C for 15 s and 72°C for 20 s. A final melt-curve step was included post-PCR (ramping from 72°C–95°C by 1°C every 5 s) to confirm the absence of any non-specific amplification. The efficiency of PCR for each primer pair was assessed using a serial dilution of 100 ng to 0.01 ng of cDNA. Each qRT-PCR experiment consisted of three independent biological replicates with three technical replicates for each. Data were analysed according to the ΔΔCT method [37], using the geometric mean of two selected housekeeping genes (*para* which encodes the voltage gated sodium channel, and *EF1a* which encodes the elongation factor 1-alpha) for normalisation according to the strategy described previously [38].

### Determination of P450 gene copy number by quantitative PCR

Quantitative PCR was used to determine *CYP4G61* gene copy number as described above but using genomic DNA (from strains TV1, TV8 and TV8pyrsel) as the template. For this, DNA from individual adult haploid male whiteflies was extracted using DNAZOL® (Invitrogen) at one tenth scale of the manufacturer's protocol and using RNase A to remove contaminating RNA. The DNA was then diluted to 2.5 ng/µl and 4 µl used in RT-PCR as detailed above. Data were analysed according to the ΔΔCT method [37] and normalised independently using two housekeeping genes, *para* (present in a single copy in insects as revealed by several genome sequencing projects [34]), and elongation factor 1-alpha (present in two copies in Hymenoptera and Diptera but in a single copy in most other insects [35]).

### Amplification of full length cDNA from *CYP4G61*

To verify the assembly, the full length coding sequence of *CYP4G61* was amplified by nested PCR using primers cyp4g61-f1 and cyp4g61-r1 in a primary PCR reaction, followed by cyp4g61-f2 and cyp4g61-r2 in a secondary reaction. Sequencing was performed using primers cyp4g61-f2, cyp4g61-f3, cyp4g61-f4, cyp4g61-f5 and cyp4g61-r2, cyp4g61-r3, cyp4g61-r4, cyp4g61-r5 (Table S3). PCR reactions (20 µl) contained 4 µl of cDNA (10 ng), 12.5 µl DreamTaq® Green DNA Polymerase (Fermentas), 15 pmol of each primer, and RNase free water. The cycling conditions were 95°C for 2 min, followed by 30 cycles of 95°C for 30 s, 50°C for 30 s and 72°C for 4 min with a final extension of 72°C for 5 min. PCR fragments were purified using the Wizard® SV Gel and PCR Clean-up System (Promega) according to the manufacturer's protocol and sent to Eurofins MWG (Germany) for direct sequencing.

### Sequence analysis

Molecular mass and isoelectric point were predicted by Compute pI/Mw tool (http://us.expasy.org/tools/pi_tool.html). The N-terminal transmembrane anchor of the CYP4G61 protein was predicted by the TNHMM Server v.1.0 (http://www.cbs.dtu.dk/services/TMHMM/). DNA and predicted protein sequences were assembled, analysed, and aligned using the Vector NTI Advance 10 package (Invitrogen). The full length sequence of the *CYP4G61* gene in this study was identified and manually curated in the recent 454-based transcriptome study of *T. vaporariorum* and it was named by David Nelson (Department of Molecular Science, University of Tennessee, Memphis) in accordance with the P450 nomenclature committee convention [21], [39]. Substrate recognition sites (SRS) were predicted by aligning the CYP4G61 protein with other P450 proteins where SRS positions were known [40].

## Supporting Information

Figure S1 **Volcano plot for the** ***Trialeurodes vaporariorum*** **microarray.** Genes meeting a p value cut-off of 0.01 and showing a transcription ratio >2 fold in either direction were considered to be differentially transcribed between the two strains and here are represented by dark dots.
(TIF)

Figure S2 **Amino acid alignment of two translated contigs (4648 and 21292) that they are coding for the full length** ***CYP4G61*** **gene.** Silent SNPs are marked in yellow coloured boxes and amino acid substitutions in green boxes.
(TIF)

Figure S3 ***CYP4G61*** **nucleotide sequences for the strains TV1 (assembled from 454 reads for this strain) and TV8pyrsel (identified by direct cDNA sequencing of this strain).**
(TXT)

Table S1 Genes identified by microarray analysis as significantly differentially transcribed between the pyriproxyfen resistant strain TV8pyrsel and the susceptible TV1. Here, the full list of these genes, along with probe name, p-value, fold-change and log2 fold-change, as well as a description based on the closest BLAST hit are detailed.
(XLS)

Table S2 Probe IDs, corresponding contigs and closest BLAST hits in the NCBI nr database for each unique contig. (Note that only the contigs that returned a BLAST result are shown).
(XLS)

Table S3 Sequences of primers used in this study. Primer sequences are listed along with the purpose for which they were designed.
(XLS)
