# Supplementary figures and images for: Over-Expression of a Cytochrome P450 Is Associated with Resistance to Pyriproxyfen in the Greenhouse Whitefly Trialeurodes vaporariorum
Source: PLoS One. 2012 Feb 8;7(2):e31077. doi: 10.1371/journal.pone.0031077 (PMC3275616; doi:10.1371/journal.pone.0031077)

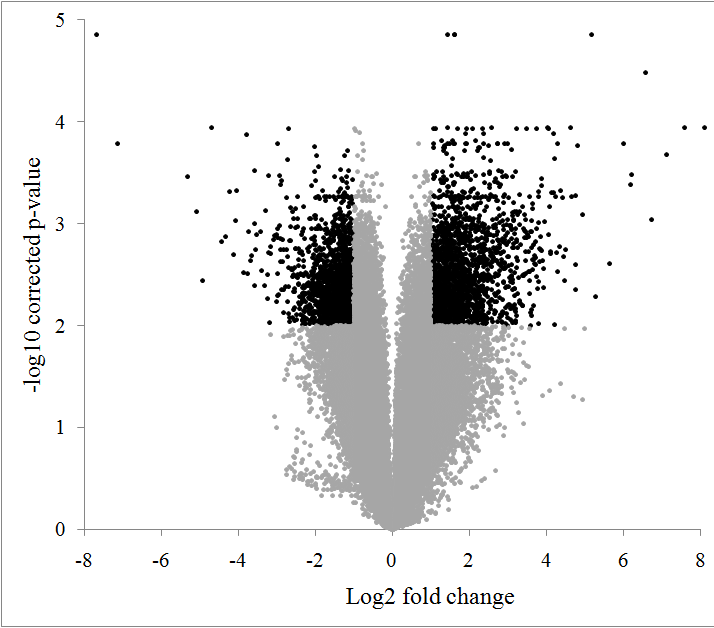

Supplement: Figure S1 — Volcano plot for the Trialeurodes vaporariorum microarray. Genes meeting a p value cut-off of 0.01 and showing a transcription ratio >2 fold in either direction were considered to be differentially transcribed between the two strains and here are represented by dark dots. (TIF) [file pone.0031077.s001.tif]

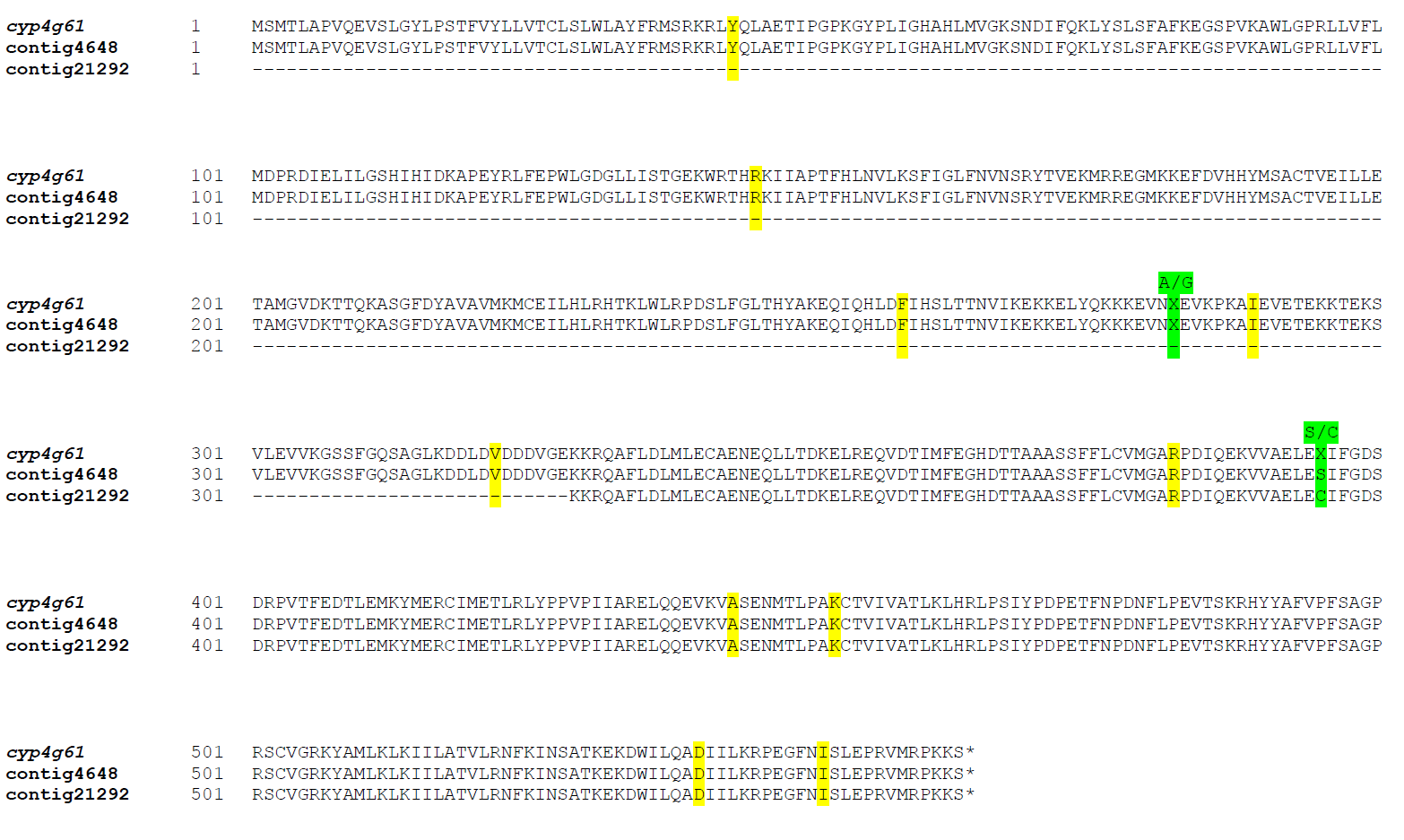

Supplement: Figure S2 — Amino acid alignment of two translated contigs (4648 and 21292) that they are coding for the full length CYP4G61 gene. Silent SNPs are marked in yellow coloured boxes and amino acid substitutions in green boxes. (TIF) [file pone.0031077.s002.tif]
